# Mitochondrial Genome Sequence of the Land Snail *Oreohelix idahoensis*

**DOI:** 10.1128/MRA.01693-18

**Published:** 2019-08-15

**Authors:** T. Mason Linscott, Christine E. Parent

**Affiliations:** a Department of Biological Sciences, University of Idaho, Moscow, Idaho, USA; b Institute for Bioinformatics and Evolutionary Studies (IBEST), Biological Sciences, Moscow, Idaho, USA; Vanderbilt University

## Abstract

We announce the complete mitochondrial genome sequence of Oreohelix idahoensis, a threatened land snail endemic to the Pacific Northwest of the United States. The circular genome is 14.2 kb and contains 13 protein-coding genes, 2 rRNA genes, and 21 tRNA genes.

## ANNOUNCEMENT

Mountain snails (Oreohelix) are the most diverse group of land snails in North America (79 species) (1), and Oreohelix spp. are the dominant malacological fauna of the Rocky Mountains (2). While the genus is widely distributed, most species in this group are endemic to single mountains or individual limestone outcrops within a mountain. The ecological specialization of these snails to small islands of limestone in montane ecosystems makes them vulnerable to extirpation, and many species are considered endangered or highly threatened by the International Union for Conservation of Nature and Natural Resources (IUCN; https://www.iucnredlist.org/) and local state governments. However, conservation officials are hesitant to develop conservation plans for many *Oreohelix* species, as recent genetic studies indicate that there is a lack of phylogenetic evidence for many existing taxonomic units (3) and the possible existence of cryptic species (4). These findings suggest that our understanding of oreohelicid diversity is limited and highlight the need for genomic resources to unravel species relationships in this group so that conservation plans can be developed based on robust species relationships.

To address this need, we sent a tissue sample of a single Oreohelix idahoensis snail collected from Lucile, ID, to the HudsonAlpha Institute for Biotechnology (Birmingham, AL) for total genomic DNA isolation and library preparation. DNA was extracted using a MagAttract high-molecular-weight DNA kit from Qiagen (product number [PN] 67563), and a 10× linked read library was prepared using a Chromium v.2 genome reagent kit (10xGenomics; PN 120258), with 2.5 ng as the starting material. The linked-read library was then sequenced on three HiSeq X 150-bp paired-end lanes, resulting in 50.2× coverage of the genome.

Following sequencing, the 10× barcode information was trimmed from the raw reads using Cutadapt (v.1.18) (5). All analyses were done using default parameters, unless otherwise stated. These trimmed reads were then assembled using NOVOPlasty (v.2.7.2) (6), which uses reference-seeded, iterative assemblies. We used a previously sequenced cytochrome oxidase subunit 1 (COI) gene of *O. idahoensis* (NCBI accession number MK263340) as the starting seed.

After assembly, we oriented and linearized the *O. idahoensis* mitochondrial genome with all other available stylommatophoran and systellommatophoran mitochondrial genomes (37 species) using the cyclic gap-free alignment tool MARS v.1.0 (7) and then aligned sequences using MAFFT (v.7.307) (8). The genome was then annotated with the MITOS Web server (v.1.0) (9), and annotations were confirmed after visually comparing BLAST alignments of the genome to all existing stylommatophoran and systellommatophoran genomes. We then constructed a maximum likelihood phylogeny using the aligned sequences with RAxML (v.8.2.9) (10), specifying a GTRGAMMA model of nucleotide evolution and evaluating nodal support by performing 100 bootstrap replicates, to determine the phylogenetic placement of *O. idahoensis*.

**FIG 1:**
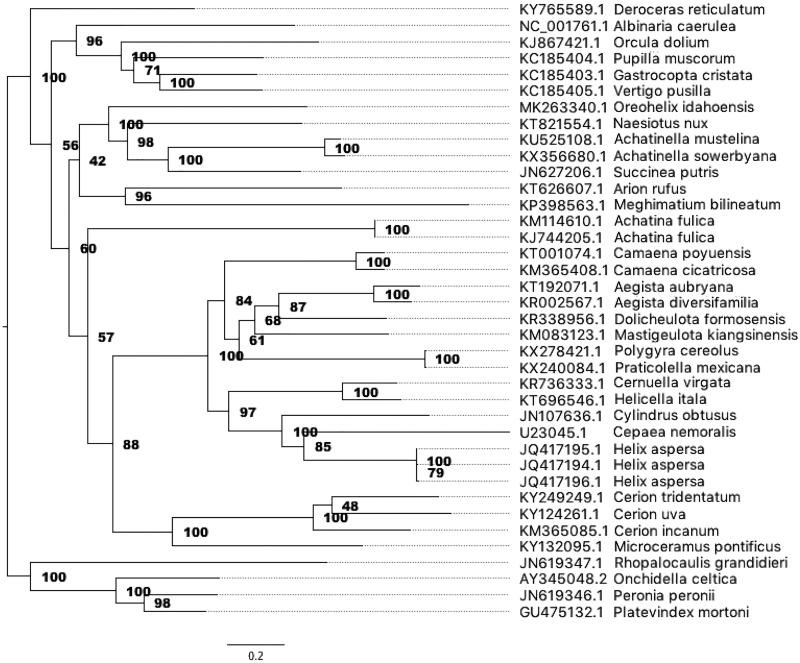
Phylogeny of 34 stylommatophoran and 4 systellommatophoran mitochondrial genomes. Node values indicate bootstrap support. Tip labels include the accession number and species. Branch lengths are in substitutions per site.

The mitochondrial genome of *O. idahoensis* is a circular DNA molecule of 14,213 bp. The G+C content is 25.64%. Annotations include respiratory genes (*atp6, atp8, cob, cox1, cox2, cox3, nad1, nad2, nad3, nad4, nad4l, nad5,* and *nad6*), two ribosomal genes (large and small subunits), and 21 tRNA genes. *O. idahoensis* is most closely related to a clade including achatinellid, bulimulid, and succineid species (Fig. 1).

### Data availability.

The genome sequence has been deposited in GenBank under the accession number MK290736, and the reads used for assembly are available in the SRA under the accession number PRJNA553530.
